# The impact of dimethylaminohexadecyl methacrylates on the physical and antibacterial properties of endodontic sealers

**DOI:** 10.3389/froh.2025.1524541

**Published:** 2025-01-31

**Authors:** Faisal Alharamlah, Fawaz AlTuwaijri, Haitham AlQuorain, Abdul Samad Khan, Faisal Alonaizan, Rashed Alsahafi, Michael D. Weir, Hockin H. K. Xu, Abdulrahman A. Balhaddad

**Affiliations:** ^1^ College of Dentistry, Imam Abdulrahman Bin Faisal University, Dammam, Saudi Arabia; ^2^ Ministry of Defense Health Services, King Fahd Military Medical Complex, Dhahran, Saudi Arabia; ^3^ Department of Restorative Dental Sciences, College of Dentistry, Imam Abdulrahman Bin Faisal University, Dammam, Saudi Arabia; ^4^ Department of Restorative Dental Sciences, College of Dentistry, Umm Al-Qura University, Makkah, Saudi Arabia; ^5^ Department of Biomaterials and Regenerative Dental Medicine, University of Maryland School of Dentistry, Baltimore, MD, United States

**Keywords:** antibacterial, quaternary ammonium, sealer, root canal, biofilm

## Abstract

**Objective:**

This study aims to incorporate contact-killing quaternary ammonium into two root canal sealers, AH Plus (DentSply Sirona, New York City, NY, USA) and BC (FKG, Le Crêt-du-Locle Switzerland) sealers to improve their antibacterial properties.

**Methods:**

Dimethylaminohexadecyl Methacrylates (DMAHDM) were synthesized and incorporated into AH Plus and BC sealers at 5 weight percent (wt.%). The physical properties were assessed via film thickness, flow, contact angle, and solubility. The antibacterial properties were assessed by determining the number of colony-forming units (CFUs) of *Enterococcus faecalis* and scanning electron microscopy (SEM). Two-way ANOVA and Tukey tests were used to analyze the data.

**Results:**

Incorporating DMAHDM at 5 wt.% increased the film thickness and reduced the flow of the AH Plus and BC sealers (*P* < 0.05), but the values were within clinically acceptable limits. Simultaneously, DMAHDM incorporation increased the contact angle of the sealers (*P* < 0.001). DMAHDM incorporation significantly (*P* < 0.001) inhibited the *E. faecalis* biofilms and resulted in complete eradication. In contrast, the AH Plus and BC control sealers had approximately 10^5^ and 10^4^ CFUs of bacteria, respectively. The SEM images revealed no *E. faecalis* colonies over the AH Plus sealers containing 5 wt.% DMAHDM, while the AH Plus control sealers were covered with a thick layer of biofilms.

**Conclusions:**

The results of this study suggest that DMAHDM, as a contact-killing agent, could be used as an approach to prevent endodontic reinfections.

**Clinical Relevance:**

Integrating DMAHDM into commercial sealers may enhance their antibacterial properties. These findings indicate a need for further investigation using more clinically relevant models to validate this approach.

## 1 Introduction

In the field of endodontics, eliminating bacterial biofilms within the root canal system and promoting the healing of the dental and periapical tissues is a major challenge due to the anatomical complexities of the root canal system and the resilient nature of endodontic biofilms (1, 2). The process of disinfection through chemo-mechanical methods and intra-canal medicament application presents significant challenges in preventing failure and recontamination (3–5). Numerous studies in the literature have demonstrated that bacteria can penetrate dentinal tubules to depths ranging from 200 to 1500 µm, making it difficult for traditional instrumentation and irrigation protocols to eradicate them (6–8).

The existence of *Enterococcus faecalis* in non-healing root canals has been broadly investigated, with previous studies reporting its presence in 23%–77% of failed endodontic treatment cases (6–8). Further studies have delved into the invasion of *E. faecalis* into dentinal tubules and its ability to survive extended periods inside the canal with limited nutrients (9, 10). Consequently, there is a need to explore more advanced approaches to suppress the growth of microorganisms embedded within dentinal tubules. After the root canal system is chemically disinfected, the primary purpose of using root canal sealers is to fill any irregularities or gaps between the gutta-percha and the root canal walls. This enhances adaptation and helps prevent microleakage (11). Although many root canal sealers possess inherent antimicrobial properties (12), these properties diminish once the sealers have set, potentially allowing for secondary endodontic infections if bacterial microleakage occurs. As a result, several efforts have been made to augment their antibacterial effectiveness by incorporating antibacterial agents into root canal-filling materials (13–15).

Recently, dimethylaminohexadecyl methacrylates (DMAHDM), a contact-killing quaternary ammonium compound with a 16-carbon alkyl chain, have shown significant antibacterial effects against several oral pathogens (16, 17). DMAHDM was found to be effective in eradicating the biofilms of several oral pathogens, such as *Streptococcus mutans* and *Candida albicans*, when incorporated into resin-based materials (17). However, there are limited studies investigating the impact of DMAHDM on *E. faecalis* biofilms. Therefore, the aim of this study is to explore the role of DMAHDM combined with different commercially available sealers in eliminating *E. faecalis* biofilms. We hypothesized that incorporating DMAHDM to different commercial sealers would improve their antibacterial properties without major changes in the physical properties of the modified sealers.

## 2 Material and method

### 2.1 Sample size calculation and study design

**Figure 1:**
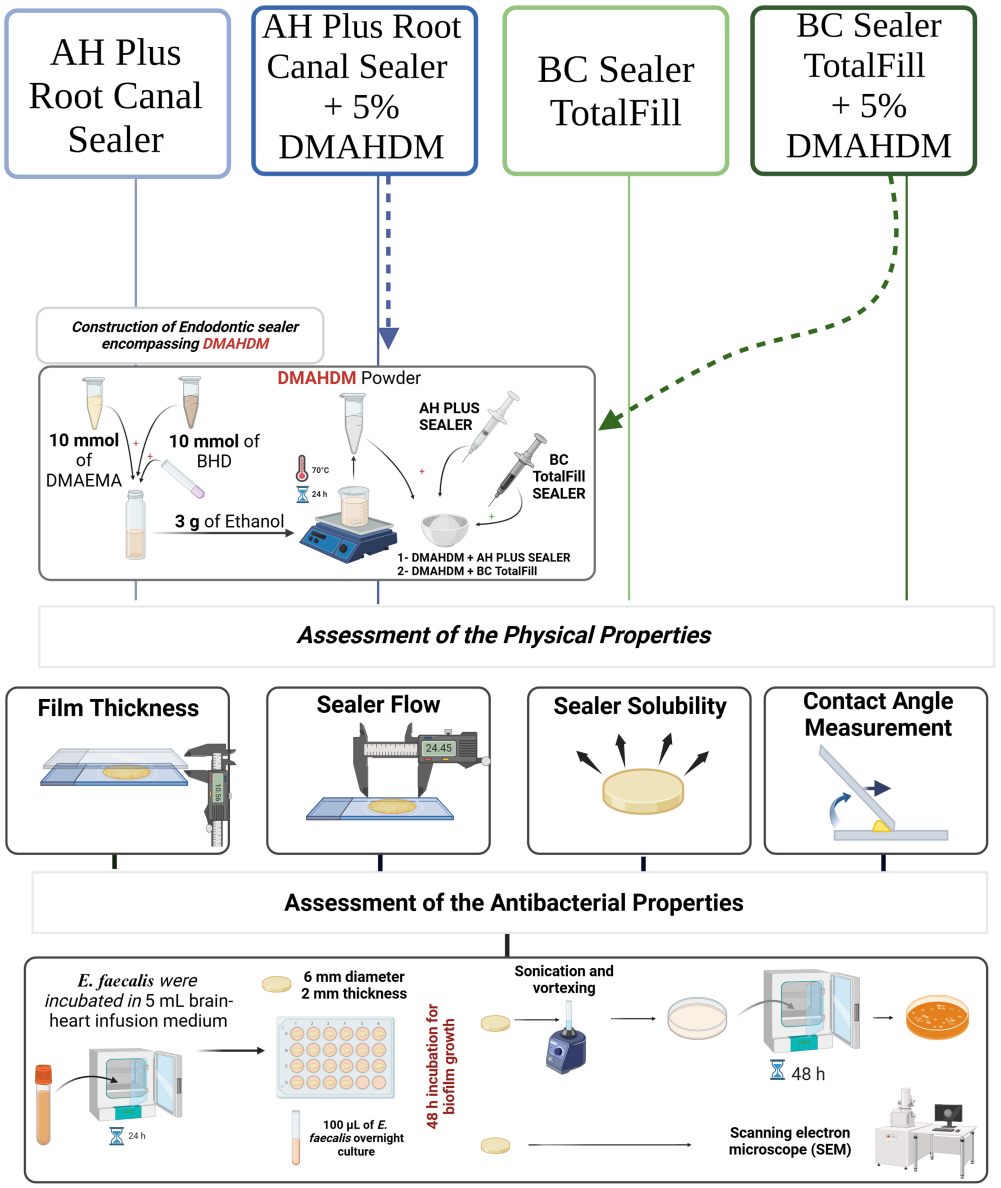
A schematic drawing showing the design of the study. DMAHDM was synthesized and incorporated into two commercial sealers at 5 wt.%. The physical properties were assessed via film thickness, flow, contact angle, and solubility. While the antibacterial properties were assessed after growing the *E. faecalis* biofilm over the sealers via determining the number of colony-forming units (CFUs) and SEM.

This study was intended to assess the physical and antibacterial properties of two endodontic sealers with and without 5 weight percent (wt.%) of DMAHDM, resulting in a total of four groups. Table 1 describes the groups that were investigated in this study. The physical properties were assessed via film thickness, flow, contact angle, and solubility, and the antibacterial properties were assessed by determining the number of colony-forming units (CFUs) and scanning electron microscopy (SEM). Based on previous studies (12, 18, 19), the minimum number of samples was three to six to assess the physical and antibacterial properties, respectively. The sample size was validated following statistical analysis, confirming its adequacy for the study. The design of the study is shown in Figure 1.

**Table 1:** The groups of sealers investigated in this study.

Group 1	AH plus root canal sealer (DentSply Sirona, New York City, NY, USA)
Group 2	5% DMAHDM + AH plus root canal sealer (DentSply Sirona, New York City, NY, USA)
Group 3	BC sealer TotalFill (FKG, Le Crêt-du-Locle Switzerland)
Group 4	5% DMAHDM + BC sealer TotalFill (FKG, Le Crêt-du-Locle Switzerland)

### 2.2 Construction of endodontic sealer encompassing DMAHDM

The synthesis of DMAHDM involved a modified Menschutkin reaction, where an organo-halide compound reacted with a tertiary amine group (20, 21). Specifically, 10 mmol of 2-(dimethylamino)ethyl methacrylate (DMAEMA) (Sigma-Aldrich, St. Louis, MO, USA) and 10 mmol of 1-bromohexadecane (BHD) (TCI America, Portland, OR, USA) were combined with 3 g of ethanol in a 20 ml vial scintillation. The resulting mixture was stirred at 70°C for 24 h. Therefore, the solvent was allowed to evaporate, resulting in the formation of DMAHDM as a solid white powder (20, 21). 5 wt.% of the synthesized DMAHDM was blended with two specific sealers as described in Table 1 by hand mixing for 20 min using a plastic instrument.

### 2.3 Assessment of the physical properties

#### 2.3.1 Film thickness

The root canal sealers were placed between two 5-mm-thick glass plates (200 ± 25 mm^2^). A 150 N weight was loaded vertically above the glass plate to ensure the sealers spread across the whole area (22). The thickness was measured 10 min after mixing using a micro-meter caliper (Dongguan Kuaijie Measuring Tool Instrument Co., Ltd., Dongguan, China). Film thickness was determined by comparing the distance between the two glass plates with and without sealers. Three measurement readings were taken for each sealer.

#### 2.3.2 Flow

The root canal sealers (0.05 ± 0.005 ml) were placed in the middle of a glass plate, 40 mm in dimension and 20 g in weight (22). A second glass plate with the same dimension and weight was placed on top of the previous one, and a 100 g load was applied for 3 min. Following the load removal, the minimum and maximum spans of the sample were measured to conclude the average span, which represents the flow of the investigated group. Each sample was assessed three times.

#### 2.3.3 Solubility

Five circular samples with a height of 2 mm and a diameter of 6 mm were fabricated per group. The sealers were allowed to set before solubility testing. The specimens were weighed (m_1_) within an accuracy of 0.001 g, immersed in deionized water, and incubated at 37° C for 14 days (23). The samples were removed at different time points and allowed to dry for 24 h using a vacuum desiccator. The samples were reweighed (m_2_) on the 3rd, 5th, 7th, and 14th day. The following equation was used to determine the solubility at each time point: (m_1—_m_2)_ m_1_ * 100%.

#### 2.3.4 Contact angle

The measurement of the water contact angle over the sealer's disks (*n* = 5) was conducted at room temperature utilizing a conventional Ramé-Hart 250 goniometer (Succasunna, NJ, USA) and advanced DROP-image advanced software. The goniometer includes a volume-controlled syringe positioned above the substrate holder, accompanied by a CCD camera that captures images of the water droplet (5 μl). The data was then processed using DROPimage advanced software. The contact angle was promptly measured within a span of five seconds.

#### 2.4 E. faecalis biofilm experiments

The sealers were fabricated with a diameter of 6 mm and 2 mm in thickness via a mylar strip covering. After complete setting, the samples were removed from the mold. The samples were sterilized by subjecting them to 70% ethanol for 15 min. *E. faecalis* (ATCC 29212) was chosen due to its association with secondary/persistent infections (7, 24, 25). *E. faecalis* were grown in brain-heart infusion broth (BHI, Sigma- Aldrich) overnight at 37°C aerobically (95% air, 5% CO_2_), following ATCC's instructions. The culture was normalized to 0.5 optical density (600 nm) (24, 25), and 100 µl of the inoculum was placed over the sealer disks for 48 h of incubation. Thereafter, the levels of biofilm formation were determined by counting the number of colony-forming units (CFUs) by serial dilution plating and by imaging the samples using scanning electron microscopy (SEM).

#### 2.4.1 Colony-forming unit (CFU)

The 2-day biofilms grown on sealer disks were transferred to vials containing 2 ml of phosphate-buffered saline. Subsequently, the biofilms were extracted by sonication and vortexing. To determine the CFUs, the biofilm suspensions were diluted in a series of steps, plated onto blood agar plates, and incubated aerobically at 37°C for 48 h. The colony count, along with the dilution factor, was determined to calculate the CFU (26).

#### 2.4.2 SEM

Only AH plus sealers with and without DMHDM were prepared for the SEM analysis. The sealers containing the grown biofilms were subjected to formaldehyde fixation. On the following day, the sealers with the biofilms were exposed to a serial dilution of ethanol followed by 100% hexamethyldisilane. SEM (Quanta 200; FEI, Hillsboro, OR, USA) images were captured with a magnification of 500–850 × at a voltage of 20 kV (26).

### 2.5 Statistical analysis

Descriptive statistics (mean, standard deviation, frequency, and percentages) used to summarize the information. Two-way ANOVA and Tukey multiple comparison tests were used to independently compare the mean values between the investigated sealers concerning their physical and antibacterial properties. A 5% significance level used in all tests. The data analyzed using Sigma Plot 12.0 (SYSTAT, Chicago, IL, USA).

## 3 Result

Both root canal sealer types and the addition of DMAHDM were significant factors pertaining to the film thickness, flow, and contact angle of the root canal sealers, while only the sealer type was a significant factor (*P* < 0.001) when the solubility was assessed (Table 2). For the antibacterial properties, the addition of DMAHDM and the use of BC sealer were significant factors (*P* < 0.001) in reducing the biofilm growth of *E. faecalis* with a significant interaction (*P* < 0.001). The interaction between the sealer type and the DMAHDM incorporation was only significant when the flow (*P* = 0.04) and contact angle (*P* < 0.001) were assessed.

**Table 2:** The impact of the root canal sealer type, DMAHDM incorporation, and their interaction on the physical and antibacterial properties of the root canal sealers.

Test		DF	SS	MS	F	*P*
Film thickness	Sealer type	1	1,365.33	1,365.33	8.51	**0** **.** **019**
	DMAHDM	1	3,745.33	3,745.33	23.34	**0**.**001**
	Sealer type × DMAHDM	1	363	363	2.26	0.171
	Residual	8	1,284	160.5		
	Total	11	6,757.67	614.33		
Flow	Sealer type	1	70.08	70.08	560.67	**<0**.**001**
	DMAHDM	1	65.33	65.33	522.67	**<0**.**001**
	Sealer type × DMAHDM	1	0.75	0.75	6	**0**.**04**
	Residual	8	1	0.12		
	Total	11	137.17	12.47		
Contact angle	Sealer type	1	11,081.88	11,081.88	2,248.97	**<0**.**001**
	DMAHDM	1	2,130.41	2,130.41	432.35	**<0**.**001**
	Sealer type × DMAHDM	1	95.56	95.56	19.39	**<0**.**001**
	Residual	28	137.97	4.92		
	Total	31	13,445.83	433.73		
Solubility	Sealer type	1	71.49	71.49	15.10	**0**.**001**
	DMAHDM	1	4.97	4.97	1.05	0.321
	Sealer type × DMAHDM	1	1.19	1.19	0.253	0.622
	Residual	16	75.76	4.73		
	Total	19	153.42	8.07		
Antibiofilm reduction	Sealer type	1	0	0	0	**<0**.**001**
	DMAHDM	1	197.27	197.27	36,938.00	**<0**.**001**
	Sealer type × DMAHDM	1	0	0	0	**<0**.**001**
	Residual	24	0.13	0.005		
	Total	27	197.39	7.31		

Bold values indicate significant difference (*P* < 0.05).

**Figure 2:**
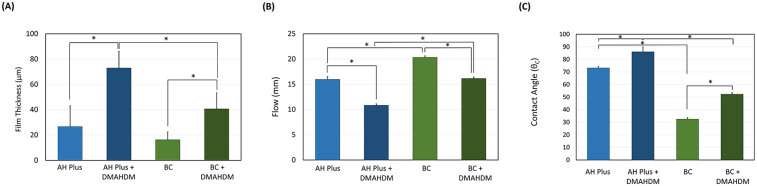
The effect of incorporating DMAHDM on the **(A)** film thickness (*n* = 3), **(B)** flow (*n* = 3) and **(C)** contact angle (*n* = 5) of the AH plus and BC sealers (mean ± SD). Stars denote statistically significant difference between the groups (*p* < 0.05).

Incorporating the DMAHDM to the AH Plus and BC sealers increased the film thickness significantly (*P* < 0.05; power of analysis = 100%) (Figure 2A). Specifically, when DMAHDM was added to the AH Plus sealer, the film thickness (73.00 ± 13.11) significantly (*P* = 0.009) increased compared to the control with no DMAHDM (26.66 ± 16.50). Similarly, in the BC sealer, adding the DMAHDM (40.67 ± 12.66) significantly increased the film thickness compared to the control with no DMAHDM (16.33 ± 6.11).

The incorporation of 5 wt.% of DMAHDM reduced the flow of the AH Plus (10.83 ± 0.28) sealer significantly (*P* < 0.001; power of analysis = 100%) compared to the parental control (16.00 ± 0.50) (Figure 2B). A similar trend was observed in the BC sealers, as the parental control (20.33 ± 0.29) revealed higher flow (*P* < 0.001) compared to the BC sealer containing DMAHDM (16.16 ± 0.30).

In general, the AH plus sealer revealed a higher contact angle than the BC sealers (*P* < 0.001; power of analysis = 100%) (Figure 2C). In addition, the incorporation of DMAHDM at 5 wt.% significantly increased the contact angle of the root canal sealers (*P* < 0.001). The interaction of the sealer type and DMAHDM incorporation was also significant (*P* < 0.001).

**Figure 3:**
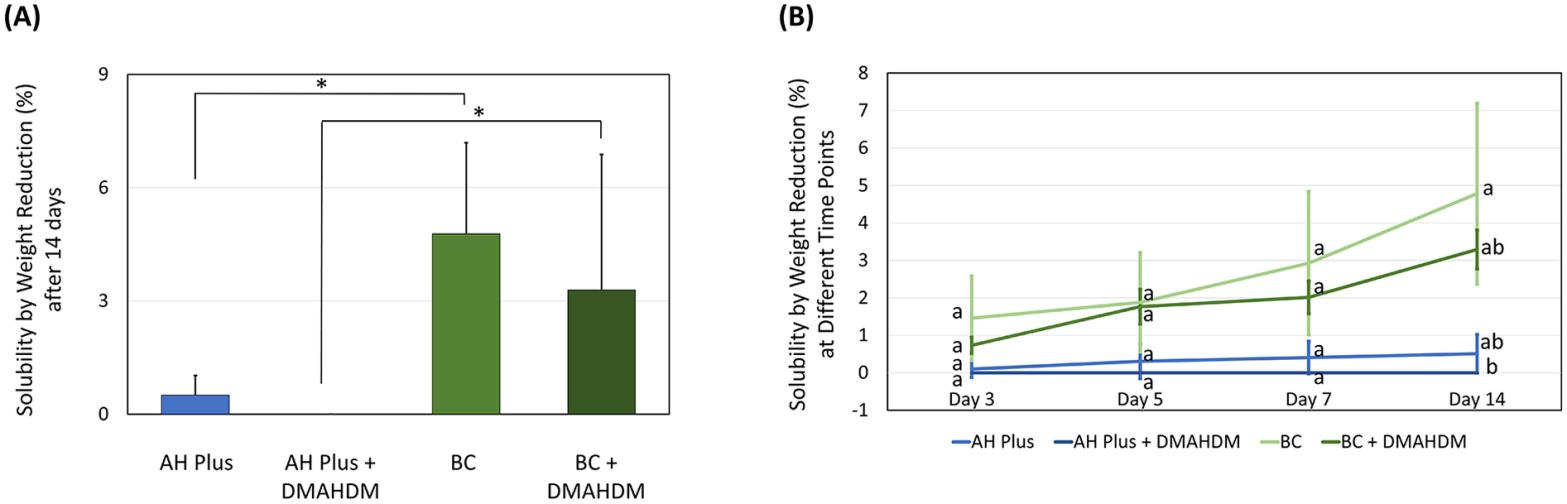
The effect of incorporating DMAHDM on the solubility of the AH plus and BC sealers (*n* = 5, mean ± SD). **(A)** The solubility of the sealers after 14 days of immersion. **(B)** The solubility of the sealers following 3, 5, 7, and 14 days of immersion. Stars and dissimilar letters denote indicate a significant difference (*P* < 0.05).

Furthermore, we observed the BC sealer had more solubility (Figure 3) than the AH Plus sealer after 14 days of immersion (*P* < 0.001; power of analysis = 100%), despite the incorporation of DMAHDM. While adding the DMAHDM reduced the sealers’ solubility, this was not significant (*P* < 0.05). The solubility by weight percentage reduction on the 3rd, 5th, 7th, and 14th day of immersion showed no significant difference at each time point except on day 14 day.

**Figure 4:**
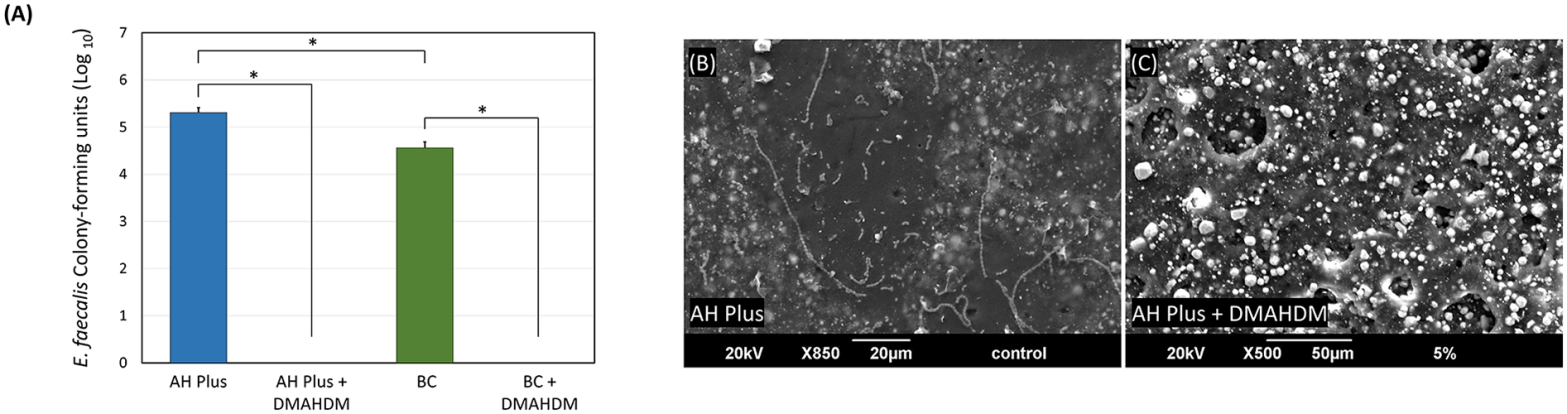
The effect of incorporating DMAHDM on the antibacterial properties of AH plus and BC sealers. **(A)** CFUs of *E. faecalis* grown over the sealer's groups (*n* = 6, mean ± SD). Stars denote statistically significant difference between the groups (*p* < 0.05). **(B,C)** Scanning electron microscope images (*n* = 2) show the *E. faecalis* biofilm growth over the AH Plus sealer with and without the addition of DMAHDM.

Incorporating 5 wt.% of DMAHDM into the AH Plus and BC sealers significantly reduced biofilm growth, resulting in complete eradication (*P* < 0.001; power of analysis = 100%) (Figure 4A). Representative SEM images reveal the same findings (Figurse 4B,C), as *E. faecalis* colonies can be visualized over the sealer without DMHADM (Figure 4B). No colonies were observed over the AH Plus sealer containing DMAHDM (Figure 4C).

## 5 Discussion

By utilizing the contact-killing properties of DMAHDM, this study successfully developed a novel material combining DMAHDM with two commercially available root canal sealers: AH Plus Root Canal Sealer by DentSply Sirona (New York City, NY, USA) and BC Sealer TotalFill by FKG (Le Crêt-du-Locle, Switzerland). The biofilm growth of *E. faecalis* bacteria was dramatically reduced by the experimental antibacterial root canal sealers that contained DMAHDM with minor changes in the physical properties of the sealers. Therefore, the hypothesis of this study was accepted.

Clinical research shows that the current chemo-mechanical disinfection techniques used in root canal treatment can be tolerated by microorganisms, which may lead to treatment failure. The Gram-positive facultative anaerobe *E. faecalis* has been identified from the root canals of patients who have not responded to treatment (25). *E. faecalis* exhibits multiple virulence factors, including the capacity to attach itself to dentin collagen, endure malnourishment, and inhibit the actions of host lymphocytes, leading to the failure of root canal treatment (24, 27). Additionally, research has shown that *E. faecalis* organized in biofilms can survive sodium hypochlorite irrigation and calcium hydroxide intracanal dressings at levels 1,000 times higher than their planktonic counterparts (24, 27). All these challenges necessitate advanced approaches to tackle endodontic pathogens during root canal treatment.

Efforts have been made to incorporate antibacterial compounds into root canal filling systems, either by immobilizing them within a polymeric matrix or by allowing the antibacterial components to be released (28, 29). Due to the materials’ intermittent release into the surrounding environment, one drawback of releasing antibacterial agents is their gradual loss of antibacterial activity (28, 29). Long-lasting antibacterial effects can be achieved by contact-killing antibacterial chemicals that are fixed within the substance and do not leak out (20). However, for these materials to succeed, the bacteria need to directly interact with their surfaces. In this study, we utilized DMAHDM as a contact-killing compound, which has demonstrated strong antibacterial properties against cariogenic biofilms when incorporated into adhesives and composites (30–32). It has been documented in this study how adding DMAHDM to a root canal sealer can help prevent *E. faecalis* biofilm formation. Two-day bacterial biofilms were evaluated in this investigation, which could be considered as immature biofilms when evaluating endodontic infections. Therefore, future investigations may adopt *ex vivo* models to test the DMAHDM-sealers inside the root canal system where the biofilms can be maintained for an elongated period, such as 21 or 28 days.

The current study employed 5 wt.% DMAHDM as a final concentration based on the findings of earlier research that showed detrimental impacts on the experimental groups’ flow and film thickness of dental materials when the DMAHDM concentration exceeds 5 wt.% (20, 30, 31). In viable *E. faecalis*, the DMAHDM sealer reduced biofilm CFU in comparison to control groups. However, to gain deeper insight into the impact of DMAHDM root canal sealer on complex biofilm structures, it is recommended that future studies employ a multi-species root canal biofilm model. This approach would enable a more comprehensive understanding of the sealer's effects on complex microbial communities within the root canal system. By utilizing a multi-species model, researchers can evaluate the sealer's efficacy against diverse microorganisms and assess its potential for disrupting biofilm formation and promoting antimicrobial activity.

The sealers’ film thickness and flow are crucial characteristics. Filling regions that are challenging to reach with instruments to stop leaks and guarantee a firm apical seal is among the most crucial roles of root canal sealers (33). To ensure proper distribution along the entire canal wall, it is essential for the sealer to possess excellent flow characteristics. Additionally, considering that sealers are more vulnerable to degradation compared to core materials, it is advisable to apply them in thin layers. However, in the present study, the introduction of DMAHDM at the investigated mass fraction negatively affected both the flow properties and film thickness parameters. Nevertheless, the values were within clinically acceptable limits.

Root canal sealers are used to create an airtight seal within the root canal system, preventing the ingress of bacteria and their byproducts. A sealer with low solubility will maintain its physical integrity over time, ensuring a durable and long-lasting seal (34). High solubility can compromise the seal's integrity, leading to microleakage and subsequent reinfection of the root canal system (35, 36). In this investigation, it was found that adding DMAHDM reduced the solubility of root canal sealers, which can reduce the risk of degradation in addition to its antibacterial properties. Besides, DMAHDM increased the contact angle when incorporated into dental sealers; therefore, less wettability is expected when the root canal sealer is unprotected to the oral atmosphere, resulting in less contamination and bacterial microleakage (20).

Despite the encouraging data found in our investigations, there are some limitations that need to be considered in future studies. First, the biofilm grown in this study was young and not mature enough to address the aggressivity of endodontic infections. Besides, this study applied a single-species biofilm model, which does not represent the actual clinical situation. As a result, future investigations may consider elongating the biofilm period, using an *ex vivo* model, and applying a multi-species biofilm. Second, this study is an *in vitro* study, and complementing the data here using a clinical translational model is highly needed to test the material inside the challenging oral environment. Third, investigating other *in vitro* physical and mechanical properties, such as elastic modulus, bonding strength, and radiopacity, is needed. Finally, there is a need for more investigation regarding how the addition of DMAHDM may affect the setting reaction of the sealers and, consequently, their performance *in vivo*.

## 5 Conclusion

This study demonstrated that sealers containing 5 wt.% of DMAHDM are effective in eradicating *E. faecalis* biofilms *in vitro*, with minor changes related to the physical properties of the sealers. These findings suggest that DMAHDM, as a contact-killing agent, could be a promising approach to prevent endodontic reinfections. However, more investigations using translational models are necessary to fully verify the effectiveness of DMAHDM against endodontic pathogens and infections.

## Data Availability

The original contributions presented in the study are included in the article/Supplementary Material, further inquiries can be directed to the corresponding author.
